# Virus-like particles that display Zika virus envelope protein domain III induce potent neutralizing immune responses in mice

**DOI:** 10.1038/s41598-017-08247-9

**Published:** 2017-08-09

**Authors:** Ming Yang, Huafang Lai, Haiyan Sun, Qiang Chen

**Affiliations:** 1 The Biodesign Institute, Arizona State University, Tempe, AZ 85287 USA; 2 School of Life Sciences, Arizona State University, Tempe, AZ 85287 USA

## Abstract

Several Zika virus (ZIKV) vaccine candidates have recently been described which use inactivated whole virus, DNA or RNA that express the virus’ Envelope (E) glycoprotein as the antigen. These were successful in stimulating production of virus-targeted antibodies that protected animals against ZIKV challenges, but their use potentially will predispose vaccinated individuals to infection by the related Dengue virus (DENV). We have devised a virus like particle (VLP) carrier based on the hepatitis B core antigen (HBcAg) that displays the ZIKV E protein domain III (zDIII), and shown that it can be produced quickly and easily purified in large quantities from *Nicotiana benthamiana* plants. HBcAg-zDIII VLPs are shown to be highly immunogenic, as two doses elicited potent humoral and cellular responses in mice that exceed the threshold correlated with protective immunity against multiple strains of Zika virus. Notably, HBcAg-zDIII VLPs-elicited antibodies did not enhance the infection of DENV in Fc gamma receptor-expressing cells, offsetting the concern of ZIKV vaccines inducing cross-reactive antibodies and sensitizing people to subsequent DENV infection. Thus, our zDIII-based vaccine offers improved safety and lower cost production than other current alternatives, with equivalent effectiveness.

## Introduction

Zika virus (ZIKV) infection in humans used to be described as a self-limiting febrile illness with symptoms of rash, headache, and myalgia. However, recent ZIKV outbreaks have linked ZIKV to the development of severe fetal abnormalities that include microcephaly and Guillain-Barre’ syndrome in adults^1, 2^. Over 1.5 million people were infected with ZIKV in Brazil in 2015 alone, and tens of millions more could be infected in the Americas in the coming years^3^. Currently, there are no licensed vaccines or therapeutics available to combat this virus. Therefore, there is an urgent call to develop effective and safe vaccines to prevent ZIKV infection.

ZIKV belongs to the genus *Flavivirus* in the family *Flaviviridae*, and is closely related to the four serotypes of dengue virus (DENV), West Nile virus (WNV), tick-borne encephalitis virus (TBEV), Japanese encephalitis virus (JEV), and yellow fever virus (YFV)^4^. Similar to other flaviviruses, the ZIKV Envelope (zE) glycoprotein is composed of three ectodomains (EDI, EDII, and EDIII)^5^ and is responsible for mediating viral assembly, attachment to cellular receptors, and the subsequent membrane fusion involved in viral entry^4^. The zE glycoprotein is also a major target of host antibody responses^4^ and its EDIII (zDIII) has been found to be targeted by several ZIKV-specific antibodies with strong neutralizing activities^6^. Since neutralizing antibodies have been shown to be correlated with protection for approved vaccines against YFV and TBEV, and to play important roles in the protection against infection by many flaviviruses including ZIKV^6–8^, zDIII is considered a prime candidate for an effective subunit vaccine due to its potential of inducing potent neutralizing antibodies.

The high degree of genetic similarity between ZIKV and DENV poses challenges for vaccine development due to the phenomenon of antibody-dependent enhancement of infection (ADE), which has been implicated for DENV infection. While antibodies generated during a primary infection of DENV are protective against the homologous serotype, these antibodies may be non-neutralizing or sub-neutralizing against a heterologous DENV serotype in a secondary infection^9^. Instead, these cross-reactive antibodies can enhance infection of the second DENV serotype in Fc gamma receptor (FcγR)-expressing cells and lead to a potentially lethal shock syndrome through ADE^10^. Since ZIKV and DENV are closely related and co-circulate geographically, any ZIKV vaccines based on common epitopes of the two viruses may have the potential to elicit cross-reactive antibodies that augment infection of DENV in vaccinated subjects when they are secondarily exposed to DENV. Indeed, a ZIKV infection can generate cross-reactive antibodies targeting the highly conserved fusion loop in EDII (EDII-FL), that serve to enhance DENV infection both in cell culture and in mice^11, 12^. Therefore, vaccine strategies based on antigens that can avoid induction of cross-reactive antibodies should also minimize the risk of ADE of DENV infections.

Recently, vaccine candidates based on inactivated virus, lipid-nanoparticle-encapsulated nucleoside-modified mRNA (mRNA–LNP), and naked or adenovirus-vectored DNA that expresses ZIKV premembrane (prM) and E protein (prM-E) were evaluated. They all have been shown to induce neutralizing antibodies that provide protection against ZIKV challenges in both mouse and rhesus monkey models^13–15^. While these developments are encouraging, hurdles remain to be overcome on the path to license these ZIKV vaccine candidates, particularly in regards to safety and cost-effectiveness.

In response, we generated a zDIII-based subunit vaccine in the form of zDIII-displaying virus-like particles (VLPs) based on the hepatitis B core antigen (HBcAg). Unlike DNA-based vaccines, there is no risk of genome insertion or associated oncogenesis by this protein-based vaccine. Furthermore, zDIII VLPs are also safer than inactivated virus and viral vector-based vaccines due to the elimination of the possibility of incomplete inactivation or unfavorable host responses to viral vectors. The use of zDIII, an antigen containing well-defined neutralizing epitopes but avoiding epitopes with ADE pathological effects, is aimed at further enhancing the safety of ZIKV vaccines while maintaining their potency. We also explored the use of an HBcAg VLP carrier to display zDIII, and plants as a production platform to increase the immunogenicity, stability, and cost effectiveness of this vaccine candidate.

## Results

### HBcAg-zDIII expression in *Nicotiana benthamiana* plants

**Figure 1:**
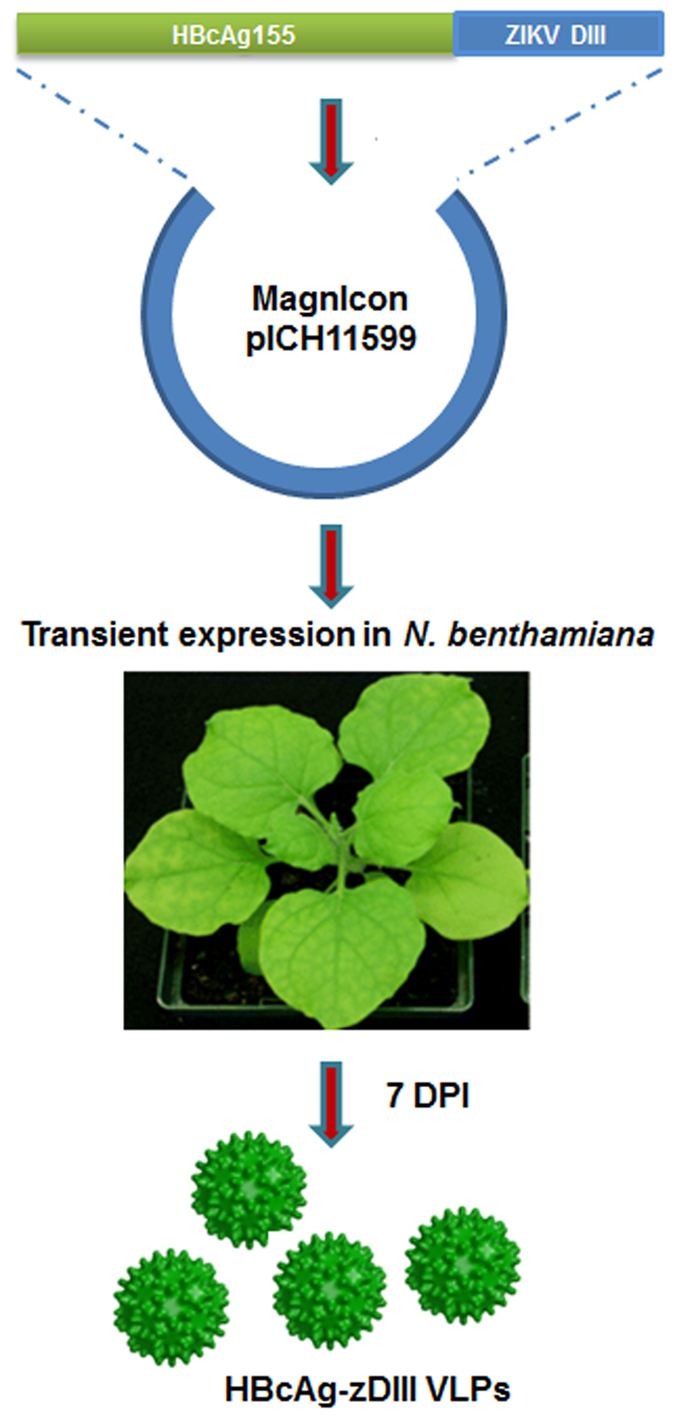
Expression of HBcAg-zDIII in *N. benthamiana plants*. The coding sequence of zDIII was fused to the 3′ end of the coding sequence of HBcAg (amino acid 1 to 155) and cloned into the MagnICON-based plant expression vector pICH11599. The *A. tumefaciens* strain that contains pICH11599-HBcAg-zDIII construct was agroinfiltrated into *N. benthamiana* leaves for transient expression. Leaves were harvested at 7 days post agroinfiltration (DPI) for HBcAg-zDIII isolation.

**Figure 2:**
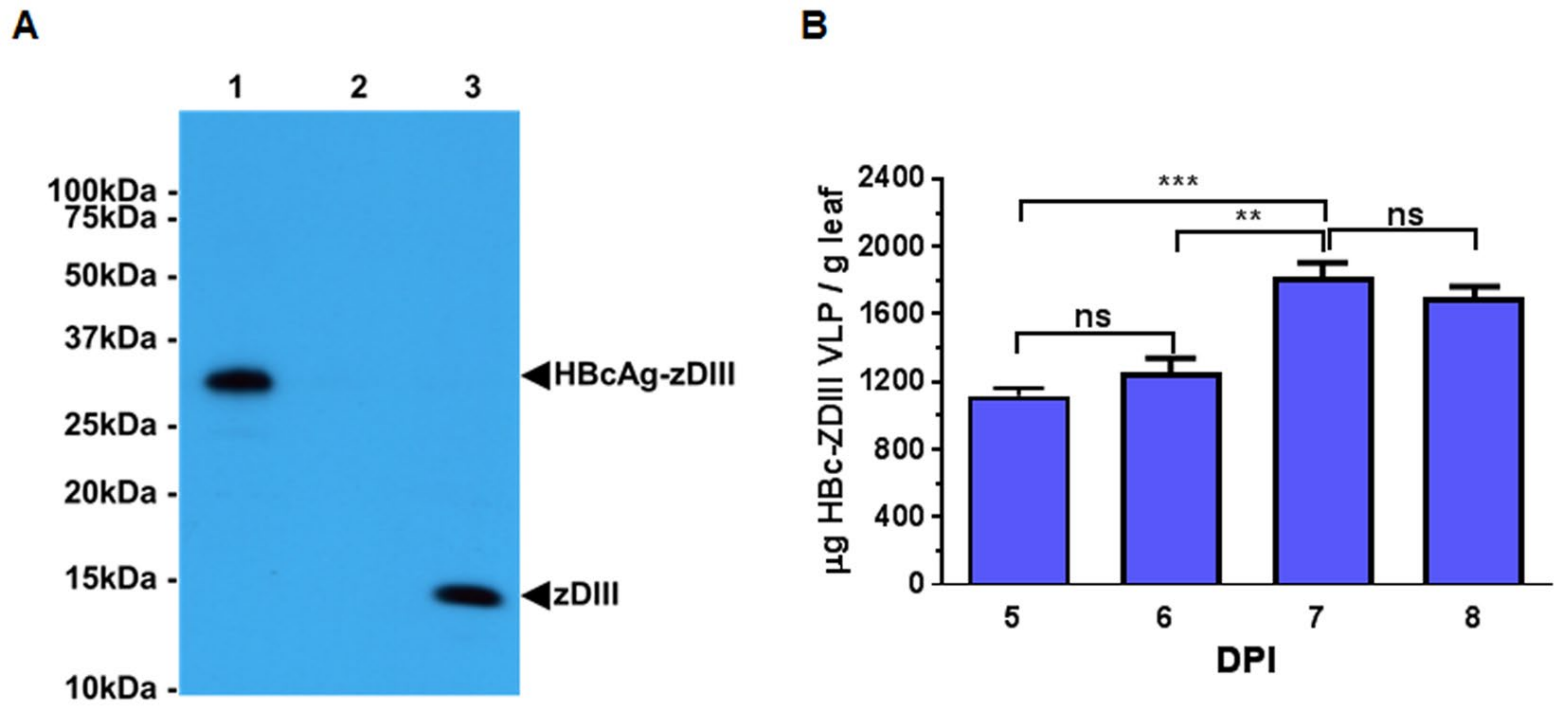
Western blot and ELISA analysis of HBcAg-zDIII. Total proteins from HBcAg-zDIII construct-infiltrated *N. benthamiana* leaves were isolated on days 5 to 8 post agroinfiltration (DPI). (**A**) Samples from 7 DPI were separated on 12% SDS-PAGE gels under reducing conditions and blotted onto PVDF membranes. The membranes were incubated with a mouse anti-zDIII antibody to detect the HBcAg-zDIII fusion protein. Lane 1, Extract from leaves infiltrated with HBcAg-zDIII construct (10 µg total protein); lane 2, Extracted from un-infiltrated leaves as a negative control (10 µg total protein); lane 3, zDIII positive control (1 µg). The full-length blot is presented in Supplementary Fig. S1. (**B**) Protein extracts were analyzed with an ELISA that detects HBcAg-zDIII. Mean ± standard deviation (SD) of samples from three independent infiltration experiments are presented. *** and **indicate p values = 0.0002 and 0.0012 of HBcAg-zDIII expression levels at 7 DPI compared to that of 5 and 6 DPI, respectively. ns = no statistically significant different (p > 0.5).

The coding sequence of zDIII was fused to the 3′ end of the coding sequence of HBcAg and cloned into MagnICON-based plant expression vectors for targeting the expression of HBcAg-zDIII in the endomembrane system of plant cells via ER^16^ (Fig. 1 **)**. The *Agrobacterium tumefaciens* strain that contained the HBcAg-zDIII construct was agroinfiltrated into *N. benthamiana* leaves. Western blot analysis was performed to evaluate the expression of HBcAg-zDIII. As shown in Fig. 2, a positive band with the predicted molecular weight for the HBcAg-zDIII fusion protein (31.7 Kda) was detected with antibodies that specifically recognize zDIII (Fig. 2A, Lane 1), indicating the expression of the fusion protein. The lack of a positive band in the negative control leaf samples (Fig. 2A, Lane 2) confirmed the specificity of the HBcAg-zDIII band. An ELISA was used to quantify the expression of HBcAg-zDIII, which showed that HBcAg-zDIII reached the highest level of production 7 days post agroinfiltration (DPI), with an average accumulation of 1,824 μg/g leaf fresh weight (LFW) (Fig. 2B). This high level of expression is similar to that previously reported for HBcAg VLPs produced in plants, representing one of the highest expression levels of recombinant proteins in plants^17^.

### Plant-expressed HBcAg-zDIII assembled into VLPs

**Figure 3:**
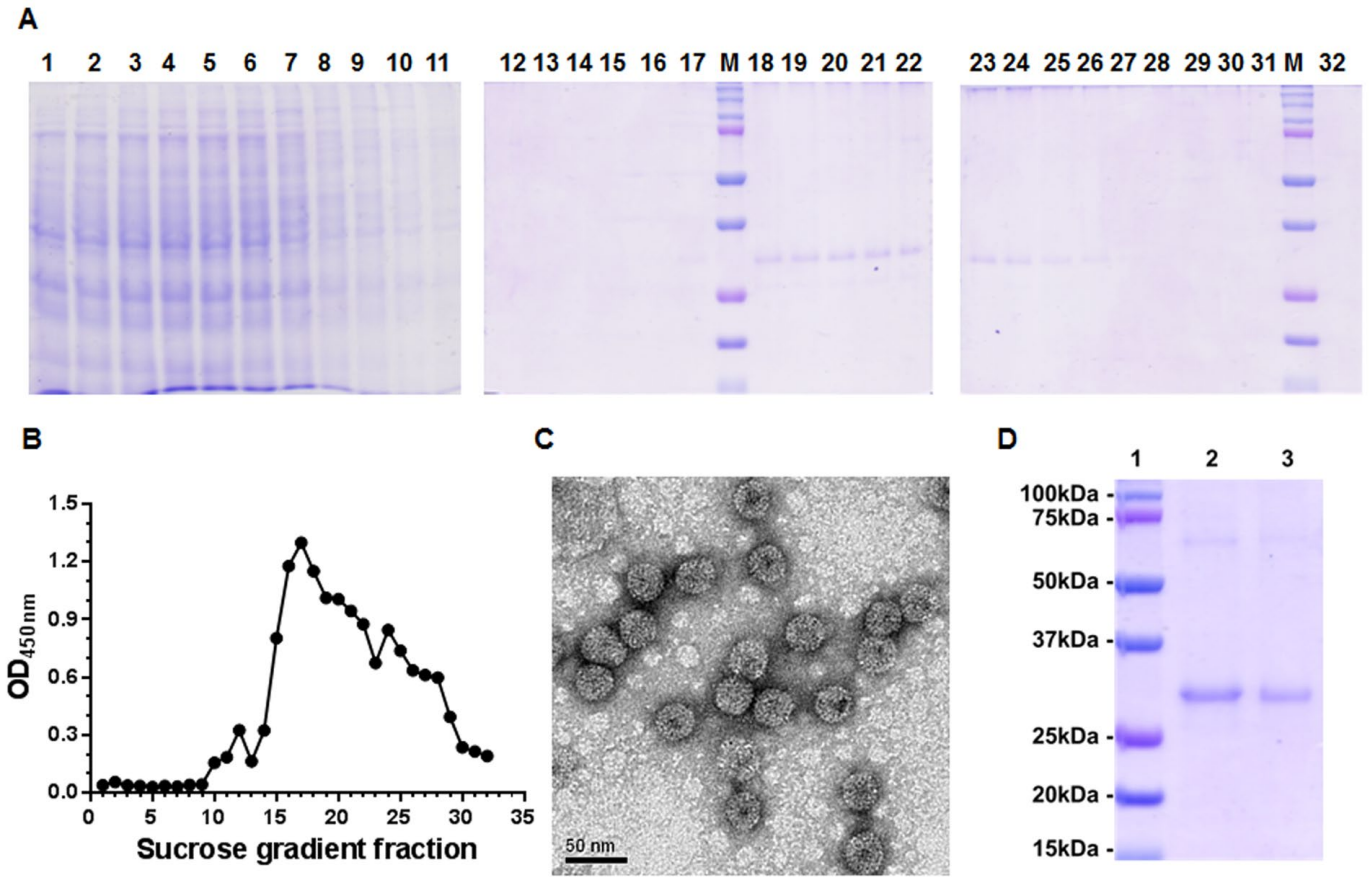
Characterization of plant-expressed HBcAg-zDIII. HBcAg-zDIII expressing leaf protein extract was subjected to a 10–70% sucrose gradient sedimentation. (**A**) SDS-PAGE analysis of sucrose gradient fractions. Sedimentation is left to right. M: molecular weight marker. Full-length gels are presented in Supplementary Fig. S2. (**B**) ELISA of sucrose gradient fractions. An anti-HBcAg antibody was used to detect HBcAg-zDIII. (**C**) Electron microscopy of HBcAg-zDIII from peak fractions of (**B**) negatively stained with 0.5% uranyl acetate. One representative field is shown. Bar = 50 nm. The full-field image is presented in Supplementary Fig. S3. (**D**) SDS-PAGE analysis of HBcAg-zDIII from peak fractions of the sucrose gradient. Lane 1: molecular weight marker; Lanes 2 and 3: 5 and 2 µg HBcAg-zDIII. The full-length gel is presented in Supplementary Fig. S4.

Clarified plant extracts were subjected to sucrose gradient sedimentation. SDS-PAGE and ELISA analysis of gradient fractions showed that HBcAg-zDIII was detected in the particulate fractions (Fig. 3A and B). When compared with HBcAg, which is known to assemble into VLPs^17^, HBcAg-zDIII was distributed in the same fractions as the parent HBcAg molecule regardless of whether anti-HBcAg or anti-zDIII antibodies were used for detection in ELISA (Fig. 3B **)**. Examination of the HBcAg-zDIII peak sucrose gradient fractions by electron microscopy conclusively confirmed the presence of typical HBcAg VLPs with a diameter of ~30 nm (Fig. 3C). The availability of an efficient purification scheme is essential for HBcAg-zDIII VLP to become a viable vaccine candidate. Indeed, the one-step sucrose gradient centrifugation process efficiently removed most plant host proteins (Fig. 3A, Lanes 1–11) and purified HBcAg-zDIII to greater than 95% pure (Fig. 3D). ELISA analysis indicated that the average recovery of HBcAg-zDIII by this process from plant extract was 64%.

### zDIII displayed by HBcAg-zDIII VLPs retained the proper folding of the native zDIII

**Figure 4:**
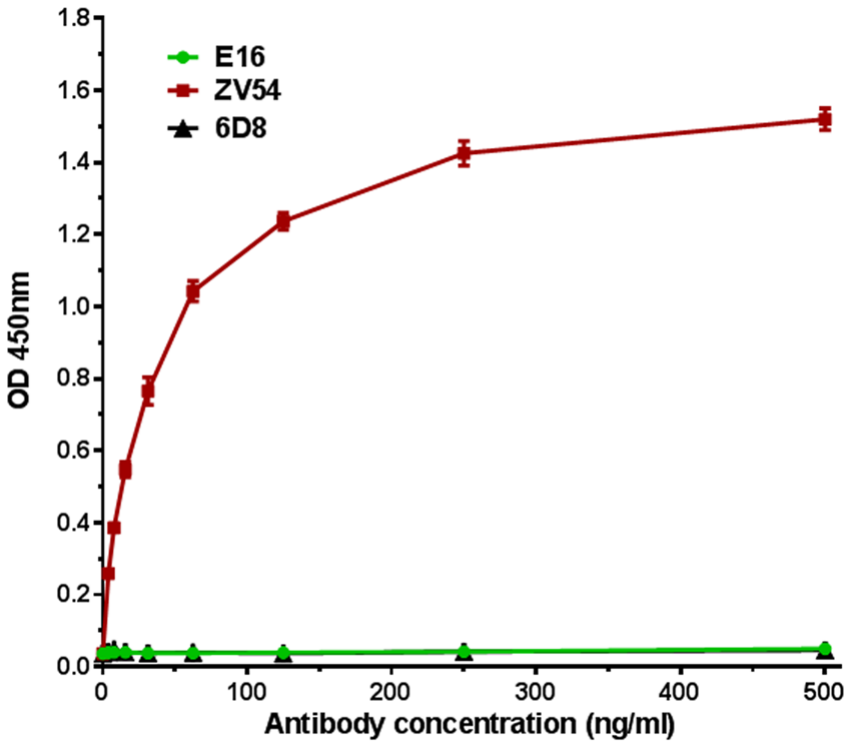
Specific binding of HBcAg VLP-displayed zDIII by monoclonal antibodies that recognize EDIII conformational epitopes. Serial dilutions of ZV54 and E16 mAbs that recognize a lateral ridge conformational epitope on EDIII of ZIKV and WNV, respectively, were incubated in microtiter wells coated with HBcAg-zDIII VLPs and detected with an HRP-conjugated goat anti-mouse IgG antibody. 6D8: an anti-Ebola isotype negative control mAb. Mean ± SD of samples from three independent experiments is presented.

To confirm the proper folding of zDIII displayed by the VLPs, the specific recognition of HBcAg-zDIII by two specific monoclonal antibodies (mAbs), i.e. ZV54 mAb and E16 mAb, was examined. E16 was generated against WNV DIII and has been shown to be WNV specific and only bind to a conformational epitope on the lateral ridge of WNV DIII^18^. In contrast, ZV54 is ZIKV specific and binds a lateral ridge conformational epitope on zDIII that consists of 4 discontinuous structural elements of the native zDIII^6^. Therefore, recognition of a recombinant HBcAg-zDIII VLP by ZV54 will be indicative of the proper folding of its zDIII moiety. Indeed, a specific and high affinity (Kd = 0.2 nM) binding of HBcAg-zDIII VLP to ZV54 was demonstrated by ELISA analysis (Fig. 4). In contrast, HBcAg-zDIII VLP did not show any binding to E16 or 6D8, an anti-Ebola IgG isotype control (Fig. 4). Thus, these results indicated that zDIII was displayed on HBcAg-zDIII VLPs in a conformation that resembles the native viral zDIII on the surface of ZIKV, suggesting the preservation of ZIKV neutralization determinants of zDIII.

### HBcAg-zDIII VLPs evoked potent and neutralizing antibody immune response in mice

**Figure 5:**
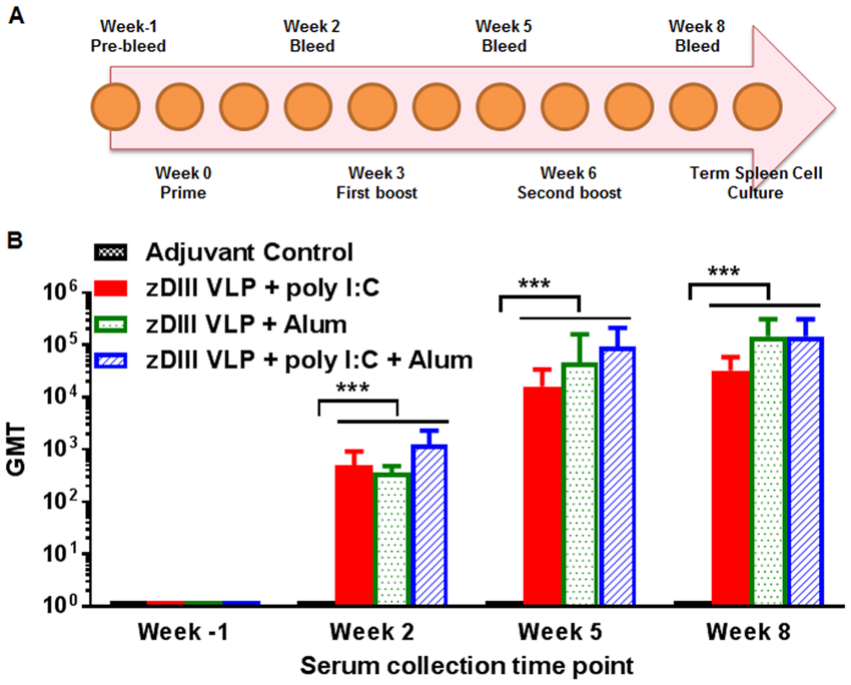
zDIII-specific IgG responses in mice received recombinant HBcAg-zDIII VLPs. C57BL/6 mice were immunized subcutaneously with three doses of HBcAg-zDIII VLP over an 8-week time period (**A**). HBcAg-zDIII VLP was injected on weeks 0, 3, and 6. The indicated adjuvant was used only in the prime injection, but not in the subsequent booster injections. Blood samples were collected on weeks -1 (preimmune bleed), 2, 5, and 8 (2 weeks after each antigen injection) and serum zDIII-specific antibody was measured by ELISA (**B**). The y axis shows the geometric mean titers (GMT) and the error bars show the 95% level of confidence of the mean. ***Indicates p values = 0.0001 to 0.0004 of HBcAg-zDIII-immunized serum compared to that of PBS + adjuvant control.

C57BL/6 mice were divided into 4 groups (n = 6 per group) and received three doses of 50 μg HBcAg-zDIII VLPs subcutaneously (Fig. 5A). Mice in group 1 (the negative control group) were injected with saline buffer (PBS) with poly I:C + alum adjuvant. Groups 2, 3 and 4 were injected with the same amount of HBcAg-zDIII VLP but with different adjuvant of poly I:C (group 2), alum (group 3) or poly I:C + alum (group 4). Adjuvant was only used in the prime injection but not in the subsequent booster injections. zDIII-specific antibody responses from individual mice were measured and Geometric mean titer (GMT) was calculated for each group. The presence of anti-zDIII IgG was not detected in sera from the control adjuvant group throughout the immunization course or in pre-immune serum samples for all groups collected prior to the first immunization (titer < 10) (Fig. 5B). The delivery of HBcAg-zDIII VLPs elicited strong zDIII-specific antibody response in all groups after the first administration (week 2, log titers > 2.55–3.1) and IgG titers reached its peak at week 5, two weeks after the first boost immunization (log titers > 4.2–4.9) (Fig. 5B). Antibody titers at week 8 (two weeks after the second boost) were similar to those of week 5 for all groups that have received HBcAg-zDIII VLPs (p = 0.44) (Fig. 5B), suggesting that the last immunization did not significantly further boost the zDIII-specific IgG response. Among different adjuvant groups, the IgG titers in the poly I:C group are significantly lower than that of the alum and poly I:C + alum groups, especially at weeks 5 and 8 (p = 0.005 poly I:C compared with alum; p = 0.003 poly I:c compared with alum + poly I:C) (Fig. 5B). The amplitude of the zDIII-specific IgG response did not differ significantly between the alum and poly I:C + alum groups throughout the immunization scheme (p = 0.87) (Fig. 5B).

**Figure 6:**
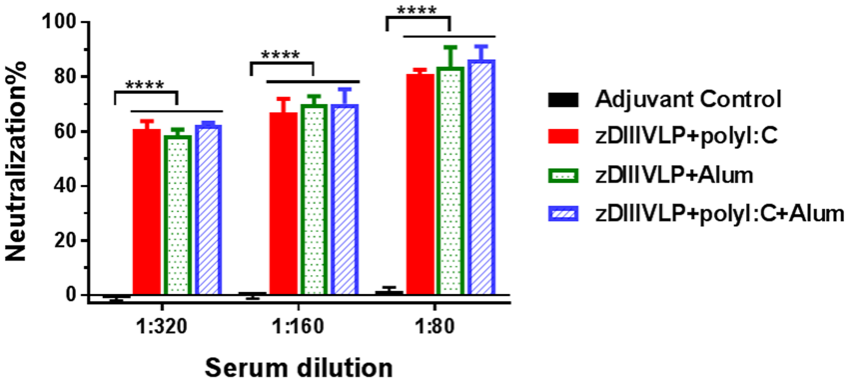
Neutralization of ZIKV by anti-HBcAg-zDIII serum. Pooled sera from week 5 of mice received PBS + Adjuvant or HBcAg-zDIII + indicated adjuvant were serially diluted and incubated with ZIKV prior to infection of Vero cells. A PRNT assay was performed as described in Materials and Methods to assess ZIKV-specific neutralizing antibodies in the sera. Mean neutralization % and SD from three independent experiments with technical triplicates for each sample are presented. ****Indicates p values < 0.0001 of HBcAg-zDIII-immunized serum compared to that of PBS + adjuvant control.

A plaque reduction neutralization test (PRNT) assay was performed to evaluate the ability of HBcAg-zDIII VLP-induced antibodies in conferring protection against ZIKV infection. As shown in Fig. 6, there was no reduction of ZIKV infection by sera from mice inoculated with PBS + adjuvant (Fig. 6). In contrast, anti-HBcAg-zDIII serum (week 5, for all three adjuvant combinations) conferred potent neutralizing effects against ZIKV infection (p < 0.0001 comparing anti-HBcAg-zDIII sera versus adjuvant alone sera) (Fig. 6). For example, greater than 60% and 80% of ZIKV infection was reduced by incubating with sera of 1/320 and 1/80 dilutions from HBcAg-zDIII VLP injected mice, respectively (Fig. 6). No significant difference of neutralization titer was observed for sera among mouse groups that received different adjuvants (p = 0.42).

### HBcAg-zDIII VLPs also elicited potent cellular immune responses in mice

**Figure 7:**
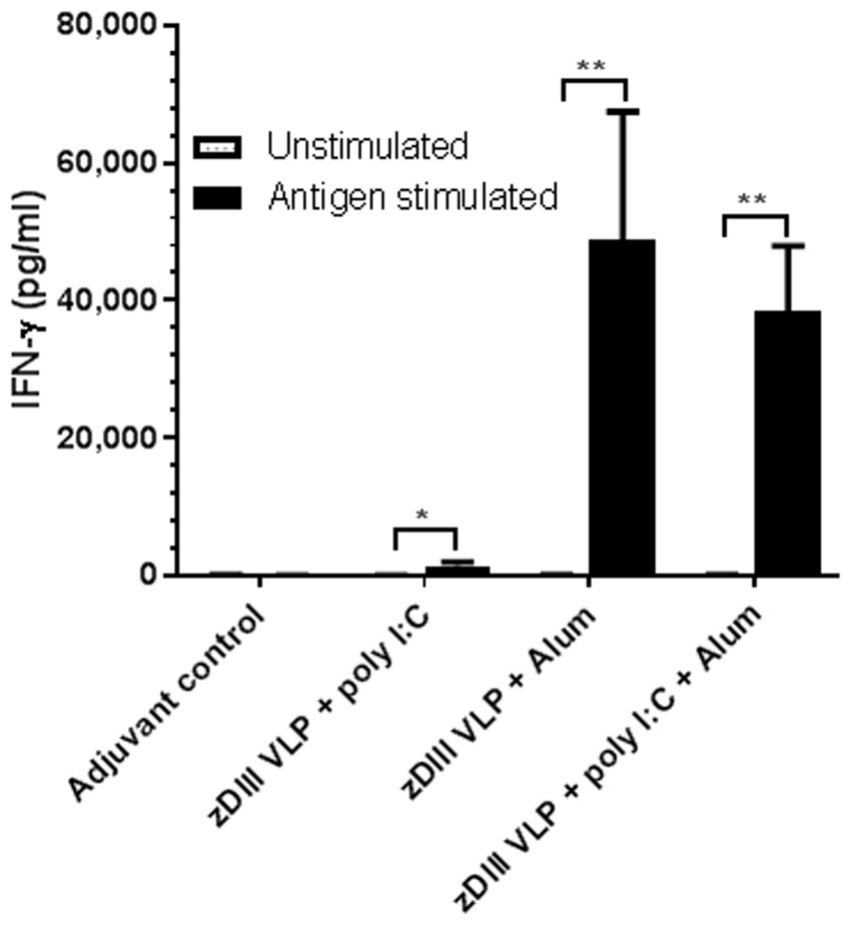
IFN-γ production by splenocytes from immunized mice. Spleen cells from mice inoculated with PBS + adjuvant or HBcAg-zDIII VLP with indicated adjuvant were stimulated with zDIII for 48 hr. The production of IFN-γ was quantitated by ELISA. Mean concentrations (pg/ml) and SD from three independent experiments with technical triplicates are presented. ** and *indicate p values = 0.0023 and 0.0286 of IFN-γ levels secreted by zDIII-stimulated splenocytes compared to that of unstimulated splenocytes.

The ability of HBcAg-zDIII VLPs in inducing cellular immune response was investigated by measuring the production of cytokines by splenocytes from immunized mice after *in vitro* antigen stimulation. The robust production of cytokines by stimulation with ConA (positive control) indicated the competency of splenocytes in producing cytokines upon stimulation *in vitro* (Fig. S5). Splenocytes of mice receiving PBS + adjuvant did not produce significant IFN-γ titers after *in vitro* stimulation with zDIII (Fig. 7). In contrast, significant levels of IFN-γ were produced by splenocytes from HBcAg-zDIII-injected mice (Fig. 7). Among the three adjuvant combinations, alum and alum + poly I:C induced strong zDIII-specific cellular immune responses as splenocytes from mice received HBcAg-zDIII VLPs with both of these adjuvant combinations producing similarly high levels of IFN-γ (p = 0.66), reaching a mean concentration of 48,736 pg/ml (alum adjuvant) and 38,496 pg/ml (alum + poly I:C adjuvant), respectively, 48hr after re-stimulation. In contrast, splenocytes from mice that received HBcAg-zDIII VLPs with poly I:C as adjuvant produced signifiantly lower levels of IFN-γ (1,522 pg/ml) (p = 0.02 compared to alum or poly I:C + alum as adjuvant). These results demonstrated that HBcAg-zDIII VLPs can induce potent cellular immune responses when appropriate adjuvants are used.

### HBcAg-zDIII VLPs circumvent induction of antibodies with ADE activity for dengue virus infection

**Figure 8:**
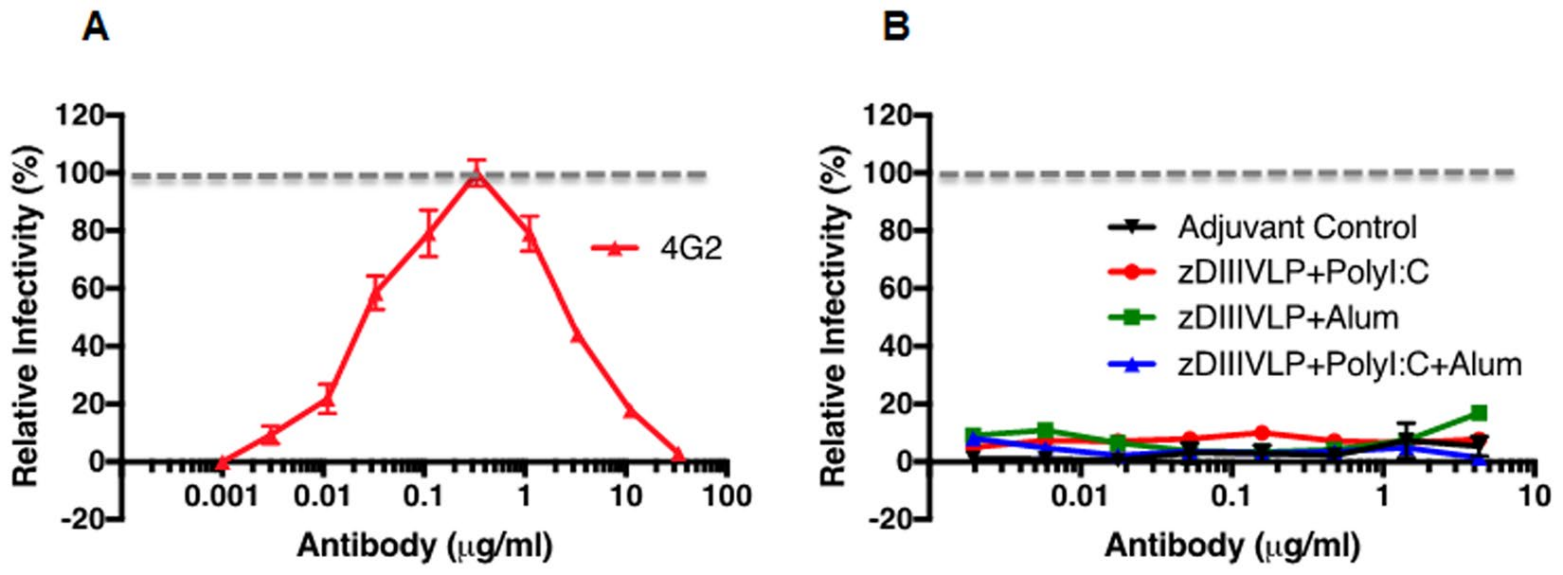
Lack of enhancement of DENV infection by antibodies in anti-HBcAg-zEDIII serum. IgGs were isolated from week 5 pooled sera of mice received PBS + Adjuvant (adjuvant control) or HBcAg-zDIII VLP + indicated adjuvant. Serial dilutions of IgGs were mixed with DENV-2 and incubated with FcγR expressing K562 cells. Forty-eight hr. later, cells were fixed, permeabilized and analyzed by flow cytometry for DENV infection. Anti-DENV-2 E mAb 4G2 was used as an ADE positive control with its maximum infectivity defined as 100%. Enhancement by IgGs from anti-HBcAg-zEDIII sera is expressed as a % relative to that of the 4G2.

One of the challenges of vaccine development for ZIKV is the risk of ADE of heterologous flavivirus (e.g. DENV) infection. As such, we investigated if our zDIII-based antigen would avoid or have diminished ability to induce cross-reactive antibodies. Dilutions of IgGs isolated from sera of HBcAg-zDIII VLP-immunized mice were incubated with DENV-2 to evaluate their ability to infect K562 cells that express the human FcγR IIA. 4G2, an anti-DENV-2 EDII mAb that is cross-reactive with E of other flaviviruses^19^, efficiently promoted ADE of DENV-2 infection of K562 cells (Fig. 8A). In contrast, IgGs isolated from HBcAg-zDIII VLP-injected mice (week 5, all three adjuvant combinations) displayed no significant ADE activity for DENV-2, similar to IgGs from the negative control mice that received PBS and adjuvant (Fig. 8B). To ensure the lack of ADE was not caused by insufficient amount of anti-HBcAg-zDIII VLP IgGs in the assay, PRNT analysis was performed to demonstrate that IgGs at high concentrations used in the assay (0.3–3 µg/ml) had neutralizing activity against ZIKV (Fig. S6). Thus, our HBcAg-zDIII VLP-based vaccine has a diminished ability to elicit the production of enhancing antibodies against DENV as demonstrated by this *in vitro* assay.

## Discussion

The worldwide ZIKV epidemic, and in particular, the link of ZIKV infection with severe abnormalities during human fetal development underscores the urgent need for potent and safe ZIKV vaccines. Recently, several types of ZIKV vaccine candidates were evaluated in animal models. These studies demonstrated that an inactivated ZIKV, a plasmid DNA, and an mRNA–LNP that all express ZIKV prM-E provided complete protection against ZIKV challenges in both mouse and non-human primate models^13–15^. Moreover, a recombinant adenovirus vector vaccine that expresses the same ZIKV prM-E protein also provided protection in resus monkeys against ZIKV challenge^14^. These studies also established that protection against various ZIKV strains including those from Brazil (Brazil ZKV2015) and Puerto Rico (PRVABC59) can be mediated by vaccine-evoked anti-zE IgG alone, and protective efficacy correlate with E-specific antibody titers (log titers > 2.35–3.2) and neutralization antibody titers (>10)^13^. While all of these vaccine candidates show great promise, there are still challenges that need to be addressed, particularly in regards to their safety and cost-effectiveness. For example, risk factors associated with incomplete inactivation of live ZIKV, unfavorable host responses to viral vectors, and the potential of ADE of heterologous flavivirus infection all spur the development of safer ZIKV vaccines, particularly for pregnant women. The wide-spread ZIKV epidemic also calls for a production platform that can rapidly produce ZIKV vaccines at a large-scale and affordable cost.

It has recently been shown that zDIII is the E protein domain that contains epitopes of potently neutralizing antibodies^6^. These anti-zDIII antibodies effectively neutralize African, Asian and American strains of ZIKV and many of them protect mice against lethal ZIKV challenges^6^. Importantly, these antibodies are ZIKV specific and, therefore, do not bind and enhance heterologous flavivirus infection through ADE. These findings and the ability of DIII of flaviviruses to independently fold into a functional domain^20^ suggest the promise of zDIII as an appealing vaccine candidate. In this study, we explored the possibility of producing zDIII in the form of a VLP and characterized its immunogenicity and safety in mice. We also tested the ability of plants as a production platform for zDIII VLP-based vaccines, aiming to address the scalability and cost issues.

Our results revealed that HBcAg-zDIII VLPs (50 µg, equivalent to 15 µg zDIII antigen) with all three adjuvant combinations elicited a potent zE-specific humoral response with zDIII-specific IgG titers (log titer) and ZIKV neutralization titers being minimally >4.2 and >320, respectively, at week 5, significantly exceeding the threshold of zE-specific (>2.35–3.2) and neutralizing antibody titers (>10) required for protection against various ZIKV strains in the mouse model. Combined with the findings that (1) the majority of epitopes that induce protective immunity against flavivirus are localized on EDIII^21, 22^ and (2) mice were effectively protected in lethal challenges from several strains of ZIKV by zDIII-specific antibodies^6^, our results suggest that zDIII-VLPs with poly I:C or alum as adjuvant can be as potent as the reported DNA or inactivated virus-based vaccines in evoking humoral response against ZIKV and may also provide protective immunity in mice.

The induction of significant levels of IFN-γ demonstrates that HBcAg-zDIII VLPs can also evoke robust cellular responses in mice. The production of high titers of IFN-γ suggests that a Th1-type or a mixed Th1/Th2 cellular immune response was elicited by HBcAg-zDIII VLPs. Generally, a Th1 or Th1/Th2 mixed response is more preferable than a Th2-type for preventing and treating viral infection^23^. Our results indicate that HBcAg-zDIII VLP-based vaccines not only can provide sterilizing immunity, but also have the potential to clear ZIKV infection. In this study, alum and poly I:C were explored as adjuvant in three formats: alum alone, poly I:C alone, and poly I:C + alum. Alum has been approved as an adjuvant for human applications and poly I:C is a synthetic analog of double-stranded RNA and a ligand of Toll-like receptor 3 (TLR3)^24^. It has been shown that TLR3 activation is important for promoting immunity and protection against flavivirus infection^25^. Our results indicate that co-delivery of HBcAg-zDIII VLPs with alum or poly I:C + alum induced significantly higher titers of zDIII-specific antibodies and cytokines than with poly I:C alone, although all three adjuvant combinations evoked ZIKV-neutralizing antibody titers that are higher than that required for protection against multiple ZIKV strains. The ability of HBcAg-zDIII VLPs with alum as adjuvant in inducing strong neutralizing humoral and cellular responses suggests the potential human application of zDIII-based VLP vaccines.

The use of zDIII-VLP based ZIKV vaccines offers several advantages over the published DNA, inactivated virus, adenovirus vector, or mRNA–LNP-based vaccine candidates. First, HBcAg-zDIII VLPs, a protein-based vaccine, will have the best safety profile compared with other vaccine platforms due to the virtual nonexistence of possible incomplete inactivation, oncogenesis by genome insertion, or unfavorable host responses to viral vectors. Additionally, we carefully chose zDIII as the antigen to target well-defined neutralizing epitopes but avoid epitopes with pathological effects. This measure is particularly crucial for vaccine development against ZIKV and other flaviviruses due to the risk of ADE. For example, individuals who were infected or vaccinated against one serotype of DENV are at a higher risk of developing dengue hemorrhagic fever/dengue shock syndrome (DHF/DSS) when they are later exposed to another serotype of DENV^9^. This enhancement of disease severity is most likely caused by ADE because many antibodies generated against the first DENV serotype are cross-reactive but sub-neutralizing against the second serotype of DENV. As a result, the new serotype forms complexes with these antibodies that bind to FcγR-bearing myeloid cells, promoting viral uptake and infection^10^. In fact, the risk of ADE by vaccination has been demonstrated by a JEV vaccine. Immunization with this vaccine has been shown to enhance the infection of all four serotypes of DENV through ADE^26^. Due to their common mosquito vectors and geographical distributions, ZIKV and DENV will continue to co-circulate in many areas of the world. Importantly, antibodies against DENV and ZIKV have been found to enhance the replication of each other *in vitro*, strongly indicating ADE may occur between these two closely-related viral diseases^11, 12, 27^. As such, minimizing the ADE risk of heterologous flavivirus infection should be an important consideration for any ZIKV vaccine development. Recent studies reveal that the FL and the adjacent region of ZIKV EDII (zDII) contain the majority of the exposed residues conserved between ZIKV and other flavivirus E proteins^28^. Likewise, the majority of DENV cross-reactive but subneutralizing antibodies in human humoral response to ZIKV E protein are targeted to epitopes on zEDII or domain I (zEDI)^12^, which is consistent with the findings in other flaviviruses^29, 30^. In contrast, antibodies against zDIII epitopes are overall ZIKV-specific, have potent neutralizing activity, and are protective against ZIKV challenge in mice^6, 12^. Notably, zDIII-specific antibodies did not show ADE activity for DENV infection while zEDI/zEDII-specific antibodies enhanced DENV infection both *in vitro* and *in vivo* ^12^. Of note, our results directly demonstrated that antibodies elicited by zDIII VLPs did not enhance DENV infection. These results indicate that our zDIII-based vaccine may provide additional safety advantages over the current candidates based on inactivated virus, DNA, or adenovirus vector, which all contain zDI/zDII and can potentially induce zDI/zDII-targeted subneutralizing antibodies and enhance DENV infection in vaccinated subjects. This safety issue is particularly important for ZIKV vaccines, where women or pregnant women make up the majority of the target population.

We choose to use HBcAg to display zDIII because numerous studies have demonstrated the immune-enhancing properties of HBcAg VLP due to the multimeric presentation of antigenic determinant on its surface and the presence of strong helper T cell epitopes within the HBcAg structure^31^. Furthermore, it has been shown that the immunodominance of some HBcAg VLP epitopes can be transferred to foreign sequences that are inserted/conjugated into these sites^32, 33^. For these reasons, HBcAg was the first VLP tested as a vaccine carrier to enhance immunogenicity of vaccines and has been shown to be effective in animal models and safe in multiple human clinical trials^31^. Like other carrier VLPs, HBcAg can also induce antibody responses to the carrier itself^34^. While it would be intriguing to investigate the extend of HBcAg carrier-specific responses elicited by HBcAg-zDIII VLPs in the future, extensive studies have shown that carrier-specific antibody responses to HBcAg are often mild and do not have significant negative influences on the immunogenicity of the foreign epitopes displayed by HBcAg VLPs^34, 35^. For example, carrier-specific anti-HBcAg antibodies were found to have minimal effect on the antigen presentation, the induction of antigen-specific antibody and cytotoxic T-cell responses, and the protective immunity of antigens that are displayed by HBcAg VLPs^34–36^. The induction of carrier-specific antibodies may also prevent the use of the vaccine in populations that have pre-exposure to hepatitis B virus. However, this challenge can be addressed by using the core antigen derived from nonhuman hepadnaviruses. For example, several core VLPs derived from the rodent hepadnaviruses have been found to be as immunogenic as the human-derived HBcAg VLPs, but are not burdened by the problem of pre-existing immunity or immune tolerance to a human pathogen^37^.

The successful production of HBcAg-zDIII VLPs in plants also helps to address the economic issues of vaccines. Since the production of plant biomass and plant-derived proteins can be scaled-up without high-capital investments of cell-culture facilities or bioreactors and expensive tissue culture media, the cost of plant-produced biologics can be greatly reduced^38^. Indeed, recent case studies have confirmed the long-held belief that plant-produced biologics is more cost effective than traditional platforms. For example, it was shown that plant-based platforms can substantially reduce the upstream production cost of biologics to as low as $1.00–2.00 per kilogram of protein^39, 40^. Our results showed that HBcAg-zDIII VLPs were accumulated rapidly at a high level of 1,824 μg/g LFW in *N. benthamiana* leaves, which is considered more than sufficient for vaccine manufacturing. This expression level under small-scale laboratory conditions can be further increased by process optimization utilizing industrial-scale plant growth conditions^41^. Plant-produced HBcAg-zDIII VLPs were readily extracted and purified from leaves with a simple, one-step purification process, which has been shown to be compliant with current Good Manufacturing Practice (cGMP) regulations and are broadly used for the production of VLP-based vaccines^41^. Thus, the quick and high-level accumulation of HBcAg-zDIII VLPs and their facile purification with high percentage of recovery indicate the potential of plants as a feasible platform for producing HBcAg-zDIII VLPs and other subunit vaccines with favorable costs and scalability. A detailed technoeconomic study is required to determine the exact cost-saving benefit of using plants for HBcAg-zDIII VLP production when manufacturing data become publicly available. However, if the cost advantage is significant, it will allow the production of affordable ZIKV vaccines for the developing world, where the majority of ZIKV cases exists.

In summary, we have demonstrated the robust production of HBcAg-zDIII VLP, its effective display of zDIII antigen and facile purification, its potent immunogenicity that correlates with protective immunity against multiple ZIKV strains, and the lack of ADE for DENV infection. To our knowledge, this is the first report of a protein-based, VLP ZIKV vaccine that induces neutralizing immunity but circumvents induction of antibodies with ADE activity for DENV infection. Altogether, our study has provided the proof-of-principle and feasibility necessary for the further development of more potent, affordable, and potentially safer recombinant protein-based subunit vaccines against the worldwide ZIKV epidemic.

## Material and Methods

### Construction of DIII expression vectors

The coding DNA sequence of ZIKV E protein of strain PRVABC59 (amino acid 1–403, Genbank Acc.No. AMC13911) was synthesized with optimized *N. benthamiana* codons (Integrated DNA Technologies, IA). The coding sequences of zDIII (amino acid 303–403 of E protein) and HBcAg (amino acid 1–155)^17^ were amplified by PCR with primer pairs (GTTTCTTACTCTCTTTGC, AGGAGCTCTCAAGATCCAAAATCCCAAGC) and (ACCATGGACATTGACCCTTAC, GCAAAGAGAGTAAGAAACACCCCTGTCCCTTCTTCG), respectively. The DNA sequence of zDIII was fused to the 3′ of HBcAg sequence by overlapping PCR with primers ACCATGGACATTGACCCTTAC and AGGAGCTCTCAAGATCCAAAATCCCAAGC. The coding sequence of HBcAg-zDIII fusion protein was then cloned into the TMV-based expression vector pIC11599 of the MagnICON system^18, 42^ (Fig. 1 **)**.

### Expression of HBcAg-zDIII in *N. benthamiana* leaves

Plant expression vectors were transformed into *A. tumefaciens* GV3101 by electroporation as previously described^43^. *N. benthamiana* plants were grown and co-agroinfiltrated with the GV3101 strain containing the HBcAg-zDIII 3′ module (pICH11599-HBcAg-zDIII) along with its 5′ TMV module (pICH20999 for ER targeting) and an integrase construct (pICH14011) as described previously^44–46^.

### zDIII expression in *E. coli*

The PCR fragment of the zDIII coding sequence was cloned into the pET28a bacterial expression plasmid (EMD Milipore, MA) with EcoRI and HindIII sites. zDIII was expressed in *E. coli* and purified using an oxidative refolding protocol as described previously^20, 47^. Purified zDIII was used in western blot and ELISA analysis.

### Extraction and purification of HBcAg-zDIII VLP from *N. benthamiana* leaves

Agroinfiltrated *N. benthamiana* leaves were harvested at 5–8 DPI for evaluating HBcAg-zDIII VLP expression. Leaves were harvested at 7 DPI for other protein analysis. Leaves were homogenized in extraction buffer (PBS, pH 5.2, 1 mM EDTA, 2 mM PMSF (Sigma, Germany)) at 1.5 ml/g LFW. The extract was clarified by centrifugation at 15,000 × *g* for 30 min at 4 °C. The supernatant was incubated for 12 hr at 4 °C, and then spun at 15,000 × *g* for 30 min at 4 °C. The supernatant was recovered and pH adjusted to 7.0. The supernatant was then subjected to a sucrose gradient sedimentation as described previously^17^. Briefly, clarified plant extracts were layered onto linear 10–70% sucrose gradients dissolved in PBS (pH 7.0). After centrifugation at 175,000 × *g* for 12 h at 4 °C, 32 fractions were collected and assayed for HBcAg and zDIII content and VLP assembly by ELISA, SDS-PAGE, and electron microscopy.

### SDS-PAGE, Western blot, and ELISAs

Samples containing HBcAg-zDIII were subjected to 12% SDS-PAGE or 4–20% SDS-PAGE under a reducing (5% v/v β-mercaptoethanol) condition as described previously^42^. Gels were either stained with Coomassie blue or used to transfer proteins onto PVDF membranes (MilliporeSigma, MA). Membranes were first incubated with a zDIII-specific mouse mAb (a gift from Dr. M. Diamond, Washington University) to detect the zDIII component of the fusion protein. Membranes were subsequently incubated with a goat anti-mouse IgG conjugated with horseradish peroxidase (HRP) (Southern Biotech, AL). Specific bindings were detected using an “ECL” Western blot detection system (Thermo Fisher, IL). Western blots and stain gels were scanned. The purity of HBcAg-zDIII VLP was quantitated using a densitometer (ChemiDoc Imager, Bio-Rad, CA) and Quantity One software (Bio-Rad, CA) by analyzing protein bands stained with Coomassie blue on SDS-PAGE as described previously^48^.

The temporal expression pattern of HBcAg-zDIII was examined by an ELISA that detects HBcAg. Briefly, plates were coated with the plant protein extract. An HRP-conjugated anti-HBcAg mAb (Abcam, MA) was used as the detection antibody. Purified HBcAg^17^ was used as a reference standard. The plates were then developed with Tetramethylbenzidine (TMB) substrate and read at 450 nm (KPL Inc, MA). HBcAg-zDIII levels in sucrose gradient fractions were also measured by the same ELISA procedure.

The specific recognition of HBcAg VLP-displayed zDIII by mAbs that bind to ZIKV DIII-specific conformational epitopes was determined by ELISA as described previously^49^. Purified HBcAg-zDIII VLP was immobilized on microtiter plates and incubated with ZV54, a mAb that only binds a lateral ridge conformational epitope on zDIII^6^. An HRP-conjugated goat anti-mouse-IgG antibody (Southern Biotech, AL) was used to detect bound antibodies. A mAb that recognizes the equivalent epitope on the DIII of WNV (E16)^50^ was used as a negative control. The binding data was used for a non-linear regression analysis using a one-site binding model (GraphPad Prism software, version 7.0, GraphPad, CA) to determine the affinity (Kd) of zDIII binding to ZV54.

ELISAs were also used to determine the titers of zDIII-specific IgG in mouse serum as previously described^43^. Specifically, microtiter plates were coated with zDIII, blocked with PBS with 1% bovine serum albumin (BSA), and incubated with a serial dilution of serum. Subsequently, the plates were incubated with an HRP-conjugated goat anti-mouse IgG (Southern Biotech, AL), washed with PBS, and developed with TMB substrate. All ELISA measurements were repeated at least three times with each sample in triplicate. Endpoint titers were defined as the highest reciprocal serum dilution that yielded an OD_450_ >2-fold over background values. GMT was calculated for each group at various time points, and was used to express the titers of the zDIII-specific total IgG.

### Electron microscopy

Purified HBcAg-zDIII VLPs were subjected to negative staining with 0.5% aqueous uranyl acetate, and transmission electron microscopy was performed with a Philips CM-12S microscope as described previously^51, 52^.

### Neutralization Assay

ZIKV-specific neutralizing antibodies were measured with a PRNT assay as previously described^53^. Briefly, ZIKV (PRVABC59, ATCC# VR-1843) was diluted in Opti-MEM medium to a working concentration of 100 plaque-forming units (PFU) per well. ZIKV was then added to the two-fold serially diluted serum and incubated for 1 hr at 37 °C. Virus/serum mixture was subsequently transferred to plates containing confluent Vero cells (ATCC # CCL-81) and incubated for 1 hr at 37 °C. After removing the virus/serum-containing medium, cells were overlaid with fresh MEM medium containing 5% FBS and 0.8% agarose (Invitrogen, CA), incubated for an additional 3 days at 37 °C, fixed in 4% paraformaldehyde (PFA, MilliporeSigma, MA), and then stained with 0.2% crystal violet. Percent (%) neutralization was calculated as: [(number of ZIKV plaque per well without anti-zDIII serum)-(number of ZIKV plaque per well of diluted anti-zDIII serum)/(number of ZIKV plaque per well without anti-zDIII serum) ×100]. Neutralizing antibody titers were expressed as the reciprocal of the highest dilution of serum that neutralized ≥50% of ZIKV. Experiments were repeated at least three times.

### Antibody-dependent enhancement assay

Sera from vaccinated mice were pooled and total IgG was isolated using IgG purification kits (GE Healthcare, PA). DENV-2 (ATCC#VR-1584) was mixed with each of eight 3-fold serial dilutions of IgG or an anti-DENV-2 E domain II mAb (4G2) (ATCC # HB-112) as positive control, respectively. The antibody-DENV-2 mixtures were incubated at 37 °C for one hour before adding to FcgRIIA^+^ K562 cells (ATCC # CCL-2243) at an MOI of 1.0. The K562 cells were then incubated at 37 °C with 5% CO_2_ for 48hr. DENV-2 infected cells were collected, fixed and permeabilized then fluorescently labeled. The percentage of infected cells was determined by flow cytometry as described previously^53^.

### Mouse immunization

All animal work was performed in accordance with the guidelines of National Institutes of Health (NIH) for the care and use of laboratory animals and approved by the Arizona State University Institutional Animal Care and Use Committee (IACUC). Six-week old female C57BL/6 mice were divided into 4 groups (n = 6 per group). Mice in group 1 received PBS with aluminum hydroxide gel (alum, InvivoGen, CA) + poly I:C (InvivoGen, CA) as a mock immunized control. Groups 2, 3, and 4 received 50 μg of HBcAg-zDIII VLP per dosage with poly I:C, alum, and poly I:C + alum as adjuvant, respectively. On Day 0, each mouse was injected subcutaneously with 100 μl of PBS or 50 μg purified HBcAg-zDIII VLP in PBS in the indicated adjuvant. Mice were boosted on days 21 and 42 using the same dosage of antigen as in the 1st immunization but without any adjuvant. Blood samples were collected from the retro-orbital vein on days 14 (week 2), 35 (week 5) and 56 (week 8) after the 1st immunization. Pre-immune serum samples were also collected on Day −7 (week −1) prior to immunization. Mice were euthanized on day 63 (week 9) and the spleens were aseptically removed for *in vitro* splenocyte cultures.

### Cytokine production in spleen cell culture

A mechanical dissociation method was used to prepare single-cell suspension of the spleens from immunized mice as published previously^47^. Splenocytes were resuspended to 5 × 10^6^ cells /ml in RPMI 1640 medium with 10% heat-inactivated FBS (Invitrogen, CA) and subsequently stimulated with 10 μg/ml of zDIII protein, T cell mitogen ConA (5 μg/ml, MilliporeSigma, MA) (positive control), and RPMI 1640 medium (negative control), respectively. The supernatant of splenocyte cultures was collected 48 hr after stimulation to determine IFN-γ production by using ELISA MAX (Deluxe Set) mouse ELISA kits (BioLegend, CA) as instructed by the manufacturer’s protocol. Experiments were performed in triplicate and repeated at least three times independently.

### Statistical analyses

GraphPad Prism software version 7.0 (GraphPad, CA) was used to perform the analysis of biochemical and immunological data. Non-linear regression analysis using a one-site binding model was used to determine the Kd of zDIII binding to ZV54. Comparisons of zDIII-specific IgG titers, cytokine concentrations, and neutralization potency between groups or between samples collected at various time points were performed by two-way ANOVA. Comparisons of HBcAg-zDIII expression levels between various days post agroinfiltration were performed with unpaired t-test. A *p* value of <0.05 indicated statistically significant differences.

### Data availability

The datasets generated by this study are available from the corresponding author on reasonable request.

## Electronic supplementary material

Supplementary information
