# Supplementary material for: Virus-like particles that display Zika virus envelope protein domain III induce potent neutralizing immune responses in mice
Source: Sci Rep. 2017 Aug 9;7:7679. doi: 10.1038/s41598-017-08247-9 (PMC5550446; doi:10.1038/s41598-017-08247-9)
Supplement: Supplementary file 1 — Supplementary information [file 41598_2017_8247_MOESM1_ESM.pdf]

**Supplementary information**

# Virus-like particles that display Zika virus envelope protein domain III induce potent neutralizing immune responses in mice

*Ming Yang[a], Huafang Lai[a], Haiyan Sun[a], and Qiang Chen[a,b,]**

The Biodesign Institute[a], School of Life Sciences[b], Arizona State University, Tempe, AZ 85287

*Corresponding author

Qiang Chen, Ph.D.

The Biodesign Institute, Arizona State University, 1001 S. McAllister Avenue, Tempe, Arizona 85287, USA

T: (480) 239-7802, F: (480) 727-7615.

Email: qiang.chen.4@asu.edu

**Legends for supplementary figures**

**Figure S1. Western blot analysis of HBcAg-zDIII expression.** Total proteins from HBcAg-zDIII construct-infiltrated *N. benthamiana* leaves were isolated and separated on 12% SDS-PAGE gels under reducing conditions and blotted onto PVDF membranes. The membranes were incubated with a mouse anti-zDIII antibody to detect the HBcAg-zDIII fusion protein. Lane 1, Extract from leaves infiltrated with HBcAg-zDIII construct; lane 2, Extracted from un-infiltrated leaves as a negative control; lane 3, zDIII positive control; Lane C1: 2-fold concentrated extract from leaves infiltrated with HBcAg-zDIII construct; Lane C2: 2-fold concentrated extract from un-infiltrated leaves; Lane C3: 2-fold concentrated zDIII positive control. A faint band under that of zDIII positive control and the expected HBcAg-zDIII was observed, suggestive of possible degradation products.

**Figure S2. Characterization of plant-expressed HBcAg-zDIII by sucrose gradient sedimentation.** HBcAg-zDIII expressing leaf protein extract was subjected to a 10-70% sucrose gradient sedimentation. Sucrose gradient fractions were analyzed by 12% SDS-PAGE and Coomassie staining. M: molecular weight marker; E: empty lane. Sedimentation is left to right.

**Figure S3. Electron microscopy of plant-produced HBcAg-zDIII.** Samples from peak HBcAg-zDIII fractions of the sucrose gradient were negatively stained with 0.5% uranyl acetate, and transmission electron microscopy was performed as described in Materials and Methods.

**Figure S4. SDS-PAGE analysis of HBcAg-zDIII from peak fractions of the sucrose gradient.** Lane 1: Molecular weight marker; Lanes 2, 3 and 4: 5, 2 and 1 µg HBcAg-zDIII; Lane E: empty lanes; Lanes P1, P2 and P3: antibody samples from an unrelated project.

**Figure S5. IFN-γ production by *in vitro* ConA stimulation in splenocytes from immunized mice.** Spleen cells from mice inoculated with PBS + adjuvant were stimulated with ConA for 48 hr. PRMI culture medium was used as a negative control. IFN-γ production was quantitated by ELISA. Mean concentrations (pg/ml) and SD from three independent experiments are presented.

**Figure S6. Neutralization of ZIKV by IgG isolated from anti-HBcAg-zDIII serum.**

IgGs were isolated from week 5 pooled sera of mice that received PBS + Adjuvant (adjuvant control) or HBcAg-zDIII VLP + poly I:C + alum. Serial dilutions of IgGs were used in a PRNT assay as described in Materials and Methods to assess ZIKV-specific neutralizing antibodies in the total IgG. Mean neutralization% and SD from three independent experiments are presented.

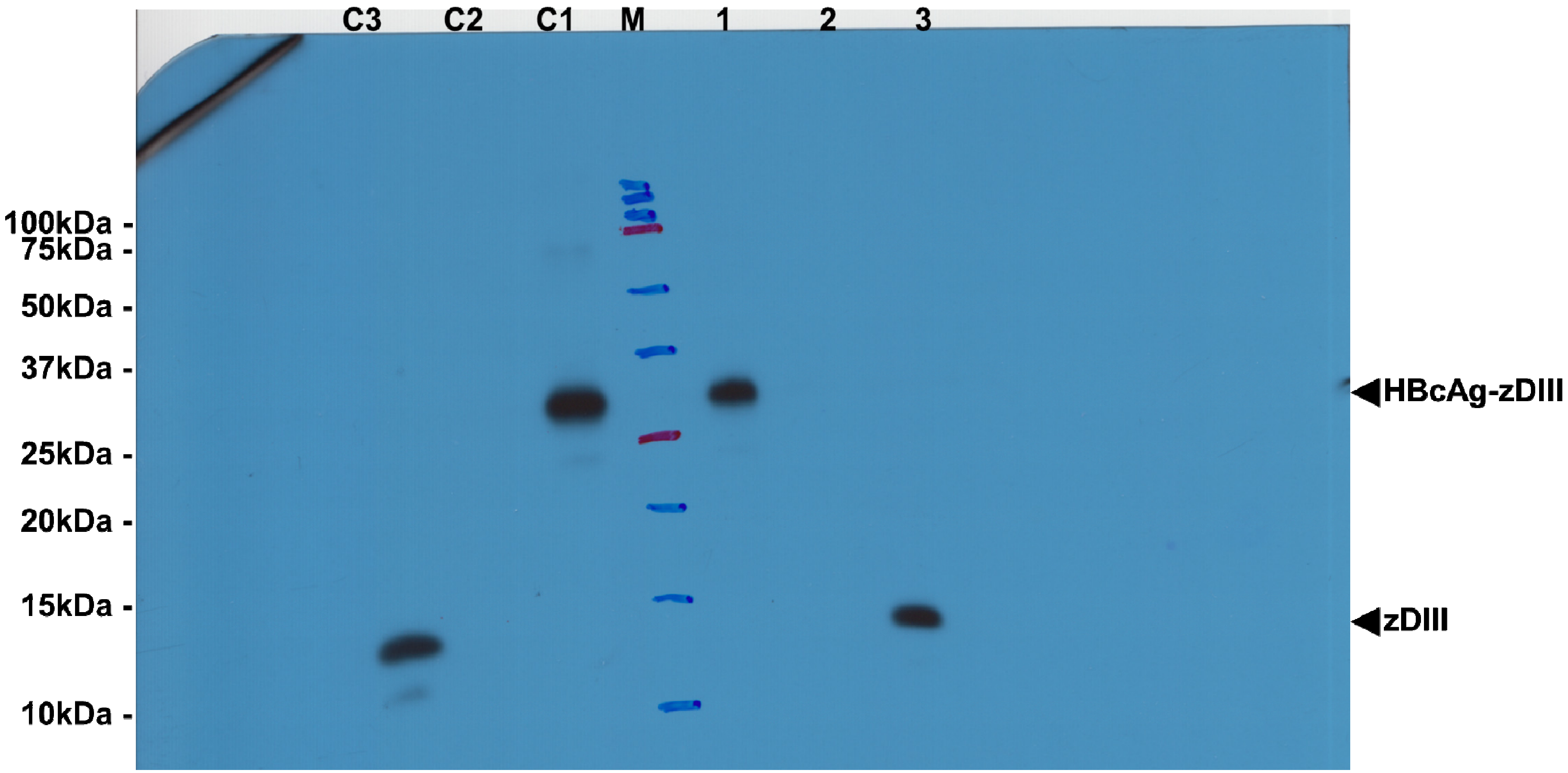


Figure S1. Western blot analysis of HBcAg-zDIII expression

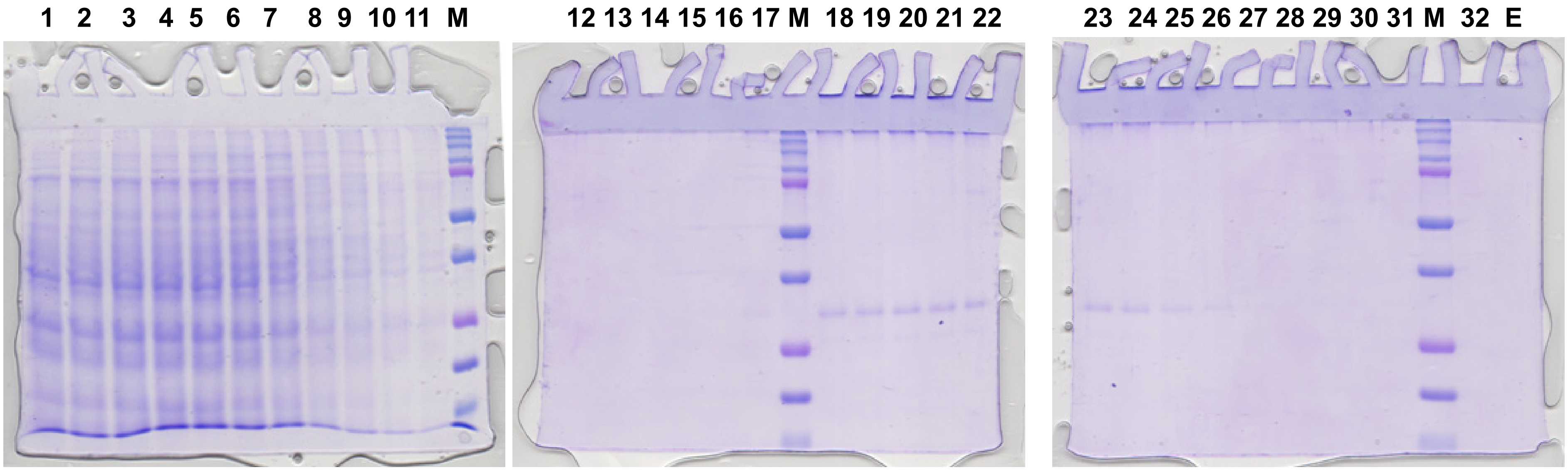


Figure S2. Characterization of plant-expressed HBcAg-zDIII by sucrose gradient sedimentation

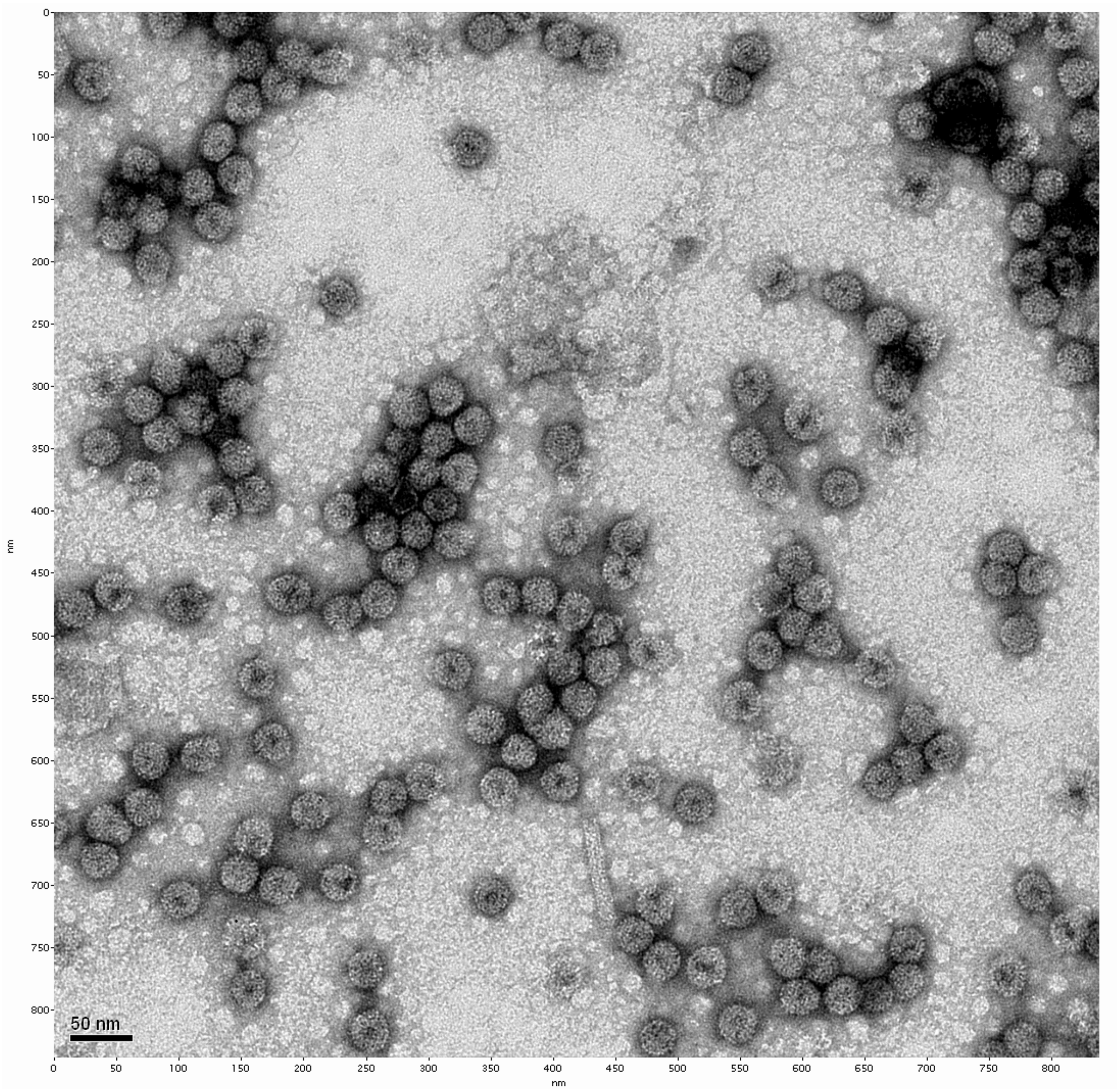


Figure S3. Electron microscopy of plant-produced HBcAg-zDIII

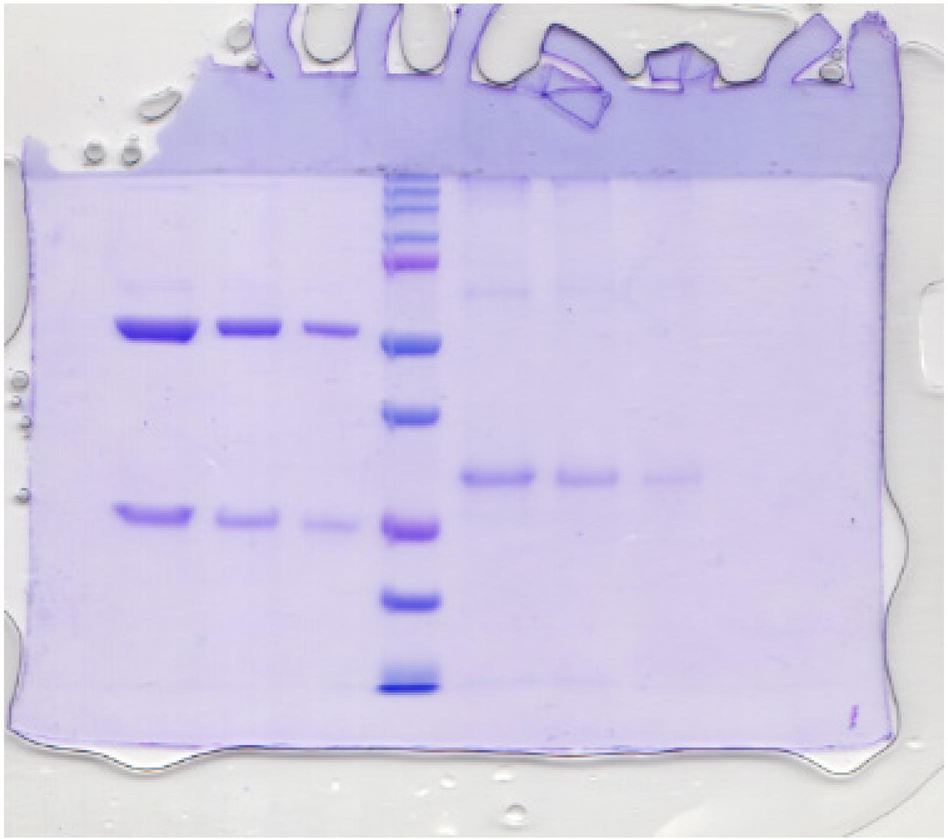


Figure S4. SDS-PAGE analysis of HBcAg-zDIII from peak fractions of the sucrose gradient

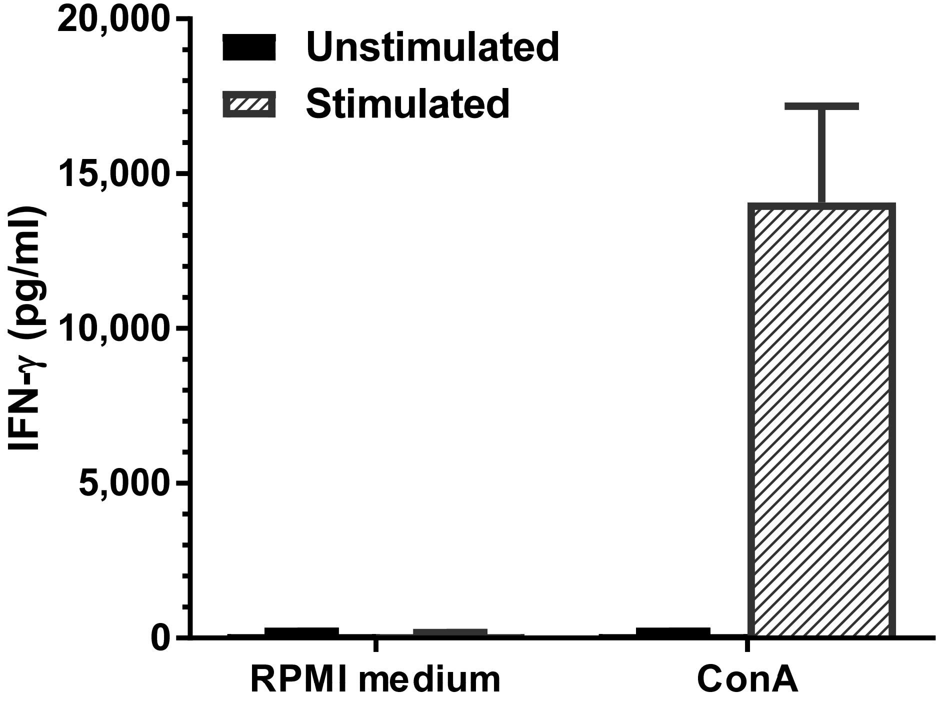


Figure S5. IFN-γ production by *in vitro* ConA stimulation in splenocytes from immunized mice.

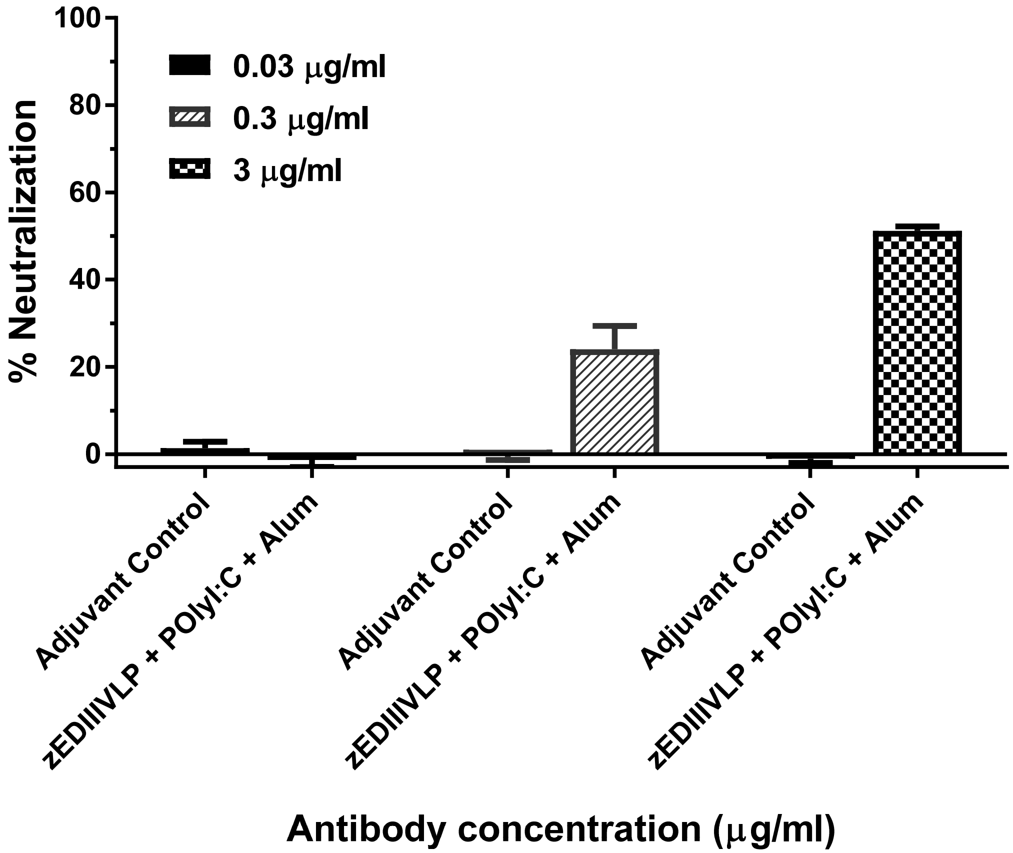


Figure S6. Neutralization of ZIKV by IgG isolated from anti-HBcAg-zDIII serum
